# Incidence and Predictors of Incidental Biochemical and Radiologic Pancreatic Alterations Following Uncomplicated ERCP

**DOI:** 10.3390/jcm12062230

**Published:** 2023-03-13

**Authors:** Millie Chau, Sunil Samnani, Fateh Bazerbachi, Anirudh Mirakhur, Yibing Ruan, Megan Howarth, Sydney Bass, Martin J. Cole, Yang Lei, Suqing Li, Christian Turbide, Rachid Mohamed, Darren R. Brenner, Steven J. Heitman, B. Joseph Elmunzer, Nauzer Forbes

**Affiliations:** 1 Department of Medicine, Cumming School of Medicine, University of Calgary, Calgary, AB T2N 1N4, Canada; 2 CentraCare, Interventional Endoscopy Program, St. Cloud Hospital, St. Cloud, MN 56303, USA; 3 Department of Radiology, Cumming School of Medicine, University of Calgary, Calgary, AB T2N 1N4, Canada; 4 Department of Oncology, Cumming School of Medicine, University of Calgary, Calgary, AB T2N 1N4, Canada; 5 Department of Cancer Epidemiology and Prevention Research, Cancer Control Alberta, Alberta Health Services, Calgary, AB T5J 3E4, Canada; 6 Department of Community Health Sciences, Cumming School of Medicine, University of Calgary, Calgary, AB T2N 1N4, Canada; 7 Division of Gastroenterology and Hepatology, Medical University of South Carolina, Charleston, SC 29425, USA

**Keywords:** ERCP, pancreatitis, lipase, radiology, health resources

## Abstract

Background: Despite post-ERCP pancreatitis (PEP) being a major focus of outcomes research in endoscopic retrograde cholangiopancreatography (ERCP), little is known regarding the frequency with which asymptomatic biochemical and/or radiologic pancreatic alterations occur in patients following ERCP. Methods: Adult inpatients undergoing ERCP were identified from a prospective ERCP registry. Patients with any abdominal pain, confirmed PEP, or pancreatitis or abnormal pancreatic enzymes preceding ERCP were excluded. Primary outcomes were asymptomatic lipase elevation on bloodwork within 24 h of ERCP or asymptomatic cross-sectional imaging findings consistent with acute pancreatitis in the absence of clinical PEP within 14 days. Multinomial logistic regression and multiple logistic regression were used to examine associations between exposures and lipase levels and between PEP or imaging findings, respectively. Results: In total, 646 and 187 patients were analyzed as part of the biochemical and radiologic cohorts, respectively. A total of 26.0% of patients had asymptomatic elevations in lipase above the upper limit of normal (ULN) within 24 h, and 9.4% had elevations >3× ULN. A total of 20.9% of patients had incidental findings of enlargement, inflammation/edema/fat stranding, peri-pancreatic fluid collections, and/or necrosis on cross-sectional imaging within 14 days. Pancreatic contrast injection was associated with higher odds of asymptomatic lipasemia (adjusted odds ratio, AOR, 7.22; 95% confidence intervals, CI, 1.13 to 46.02), as was the use of the double-wire technique (AOR 15.74; 95% 1.15 to 214.74) and placement of a common bile duct stent (AOR 4.19; 95% CI 1.37 to 12.77). Over 10 cannulation attempts were associated with the presence of one or more radiologic finding(s) (AOR 33.95; 95% CI 1.64, to 704.13). Conclusions: Significant rates of incidental biochemical and/or radiologic pancreatic abnormalities are present following ERCP. Clinicians should be aware of our findings to minimize misclassification and better direct healthcare utilization.

## 1. Introduction

Endoscopic retrograde cholangiopancreatography (ERCP) is well established as a first-line modality in the management of several categories of pancreaticobiliary disease [1,2]. ERCP is highly effective, but its performance is associated with several adverse events (AEs), with the most well-described being post-ERCP pancreatitis (PEP) [3]. A diagnosis of PEP is made when abdominal pain characteristic of pancreatitis is accompanied by pancreatic enzyme (lipase or amylase) elevation, typical imaging findings, or both [4,5,6].

However, following ERCP, patients frequently experience abdominal bloating, biliary-type and/or stent-associated pain, and/or other types of pain not related to PEP [7,8]. This is important, given that the occurrence of non-specific abdominal pain in the post-ERCP setting often prompts a reflex measurement of serum pancreatic enzymes to rule out PEP. An ensuing elevation of lipase or amylase ≥ 3 times the upper limit of normal (ULN) accompanying such non-specific pain may therefore result in a false diagnosis of PEP. The same is true regarding incidental radiographic findings typically associated with acute pancreatitis in cross-sectional imaging studies requested for unrelated reasons following ERCP. These misdiagnoses have the potential to carry with them several clinical, medicolegal, and research-related implications, including improper management and/or work-up, misclassification in administrative and/or research records, costly subsequent interventions and/or incorrect patient counseling, which can result in increased apprehension, potentially reduced adherence to follow-up recommendations, or hesitation and/or doubt when faced with the need to undergo medical procedures in the future [9].

Despite these considerations, relatively little is known regarding the frequency with which alterations in biochemical and/or imaging parameters occur in asymptomatic patients following uncomplicated ERCP. Estimates of post-ERCP pancreatic enzyme increases within hours of the procedure are available [10,11], but estimates of the proportions of patients who experience alterations on routine measurement are scarce [12] and mainly confined to randomized trials, which may not represent real-world practices and generally do not have the ability to identify truly asymptomatic patients. Estimates of proportions of patients who are observed to have typical post-ERCP pancreatic imaging findings in the absence of adverse events are altogether absent. Furthermore, little is known about the relevant patient-related and procedure-related predictors of developing these changes in biochemistry and/or imaging, and whether these predictors parallel those that are established for PEP. Thus, we performed an analysis of a prospective registry to assess the incidence and predictors of abnormal biochemical and/or radiologic changes following uncomplicated ERCP.

## 2. Patients and Methods

### 2.1. Study Design and Setting 

This study was designed and executed as an analysis of a prospective ERCP cohort study at a tertiary referral center in Calgary, Alberta, Canada [13]. All ERCPs were performed by 1 of 5 endoscopists who had individually performed over 1000 ERCPs prior to the index procedures or by advanced endoscopy trainees under their direct supervision. Institutional research ethics board approval was obtained for the prospective study (REB18-0410). All patients have routine follow-up via phone call 30 days after ERCP, with any unplanned primary care visits, emergency department visits, and/or hospitalizations captured along with all relevant associated details by a combination of patient history and review of the medical record [13]. Thus, all occurrences of suspected PEP are reviewed and (a) either confirmed as PEP and attributed a likelihood of procedural causality or (b) rejected as not meeting the definition of PEP based on a recently published classification system [4]. Furthermore, the occurrence of self-recalled intra- and/or post-procedural pain is also captured prospectively as part of the registry according to a validated scale designed to capture patient-reported experiences in endoscopy [8]. 

### 2.2. Patients, Variables, and Outcomes

All patients ≥ 18 years old who underwent ERCP at our center between 1 September 2018 (the inception of the registry) and 28 February 2022 were initially identified. Outpatients were excluded given a low likelihood of having routine bloodwork (including pancreatic enzymes) or cross-sectional imaging following their procedure. For the biochemical cohort, patients with any post-procedural abdominal pain 3/10 or greater [8], with any recalled self-reported post-procedural pain on follow-up, or with confirmed PEP were excluded from the cohort. Furthermore, any patients with acute pancreatitis (from any cause) prior to the procedure were excluded, as were patients with amylase or lipase levels measuring ≥3× ULN within 7 days preceding ERCP. Additionally, any patients without an established normal amylase or lipase (<3× ULN) prior to ERCP were also excluded given the absence of a reference point. 

At our institution, providers have various options when ordering laboratory parameters for patients regarding timing and urgency, with menu selections helping to guide decision making. One such option is a choice regarding “routine” or “morning” bloodwork. Thus, as a final exclusion criterion, any patients who had “stat” or non-“routine+morning” bloodwork drawn following the procedure were also excluded, with an electronic database filter applied to exclude such patients. In so doing, in combination with the exclusion criteria listed above, we minimized the likelihood of including any symptomatic patients in our cohort.

For the radiologic cohort, in addition to applying all exclusion criteria above, any patients undergoing imaging studies for indications of suspected pancreatitis or to elucidate other potential causes of abdominal pain following ERCP were excluded, as were patients without baseline pre-procedural imaging during the same admission indicating no abnormalities in the pancreas and patients with cross-sectional imaging studies of body parts other than the abdomen and pelvis.

As part of the ERCP registry, all relevant patient baseline demographics and comorbidities were captured, in addition to the procedural indication, the involvement of trainee(s), and prior ERCP status. In addition, several intra-procedural variables were prospectively captured by third-party observation (i.e., without endoscopist self-reporting), including: injection of pancreatic contrast, inadvertent pancreatic duct cannulation, use of the ‘double wire’ technique, performance of sphincterotomy and/or sphincteroplasty, performance of ‘pre-cut’ sphincterotomy or needle-knife papillotomy, the number of total cannulation attempts, the cannulation time and overall procedure time, and the placement of any stents (and locations and types). 

The primary outcomes were either (1) asymptomatic lipase elevation following uncomplicated ERCP or (2) asymptomatic cross-sectional imaging findings consistent with acute pancreatitis in the absence of clinical PEP following ERCP. For the biochemical cohort, all patients meeting the eligibility criteria above with routinely measured lipase measurements recorded on the day following ERCP were included and placed into categories of ‘no lipasemia’ (lipase up to and including 80, the ULN at our center), ‘lipasemia 1–2× ULN’, ‘lipasemia 2–3× ULN’, and ‘lipasemia >3× ULN’, with those with established PEP included as a comparator group. For the radiologic cohort, all patients meeting the eligibility criteria above with either computed tomography (CT) or magnetic resonance imaging (MRI) studies of the abdomen and pelvis within 14 days following ERCP were included. Incidental imaging findings that would otherwise be consistent with pancreatitis were considered, including pancreatic enlargement, edema/inflammation/fat stranding (lumped together given the overlap in terms describing a similar appearance of the gland), peri-pancreatic fluid collections, and necrosis.

### 2.3. Statistical Analyses

Descriptive statistics were presented as means with accompanying standard deviations (SD) and percentages by lipase categories and PEP, or by imaging categories. Across-group variations or associations were tested with one-way ANOVA for normally distributed data, Kruskal–Wallis test for skewed data, or chi-squared tests for categorical data. Multinomial logistic regression was used to examine the associations of the clinical and procedural variables with the categories of lipase levels. Multiple logistic regression was used to examine the associations of exposure variables with PEP or image findings. All analyses were performed using R version 4.0.2 (R Foundation for Statistical Computing, Vienna, Austria) and STATA 17.0 SE (StataCorp, College Station, TX, USA).

## 3. Results

### 3.1. Demographics and Descriptive Results

**Figure 1:**
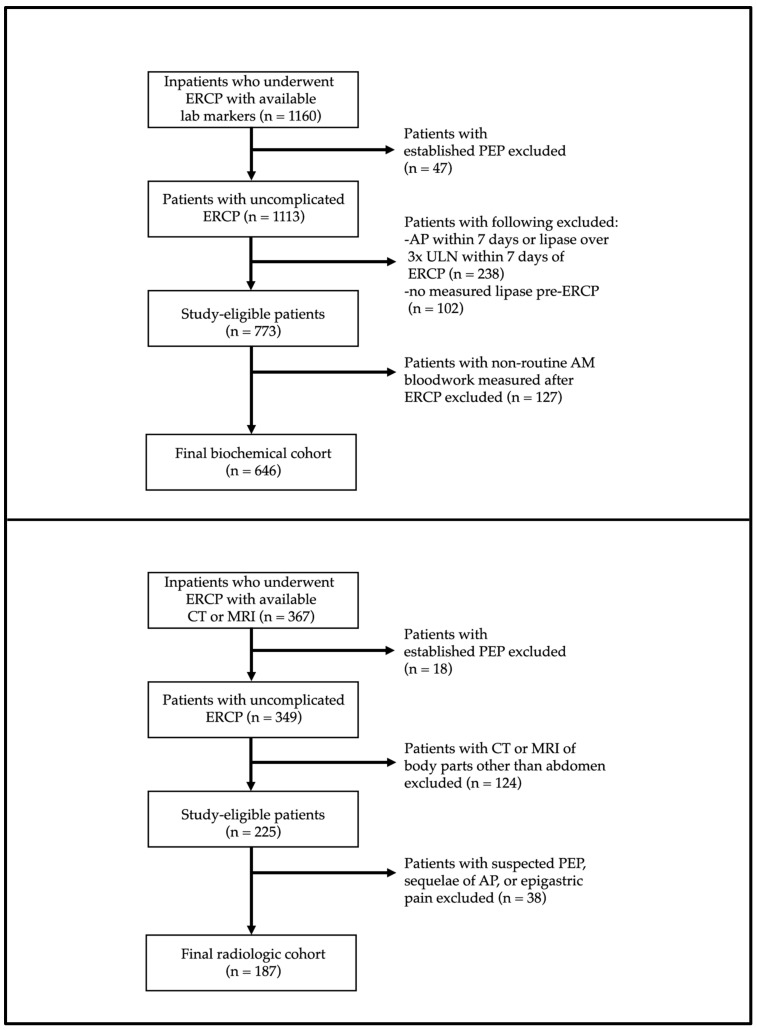
Study flowchart with exclusions for biochemical and radiologic cohorts.

A detailed study flow diagram is provided in Figure 1 that shows the number of patients excluded (with reasons) from the biochemical and radiologic cohorts. A total of 1160 and 367 inpatients were initially eligible for inclusion in the biochemical and radiologic cohorts, respectively. Forty-seven patients of the original 1160 inpatients (4.1%) were diagnosed with confirmed PEP. After applying all exclusions, 646 patients were analyzed as part of the biochemical cohort, and 187 patients were analyzed as part of the radiologic cohort. Demographic, clinical, and procedural details for the biochemical and radiologic cohorts are presented in Table 1 and Table 2, respectively.

**Figure 2:**
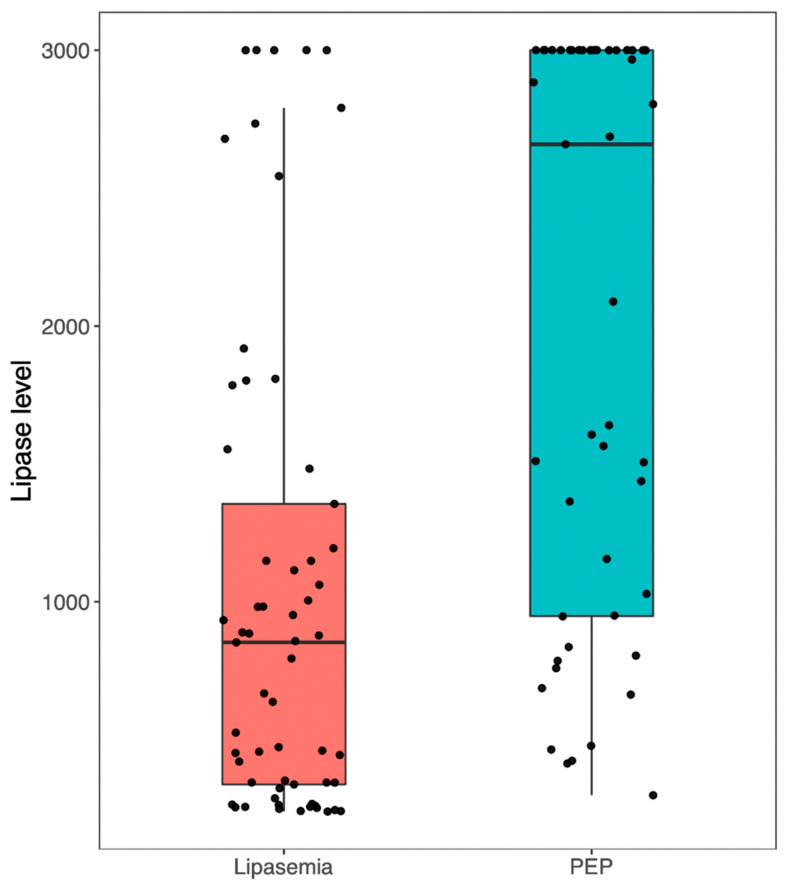
Distribution of lipase levels (measured on the day following ERCP) for patients with asymptomatic lipasemia and PEP. Highest value of lipase reported as ‘>3000 U/L’. PEP, post-endoscopic retrograde cholangiopancreatography pancreatitis.

In the biochemical cohort, 478 patients (74.0%) had no elevations in pancreatic enzymes above the ULN within 24 h, while 81 (12.5%) had elevations up to 2× ULN, 26 (4.0%) had elevations between 2 and 3× ULN, and 61 (9.4%) had elevations >3× ULN while having no abdominal pain. Boxplots demonstrating the distribution of lipase readings by category are provided in Figure 2.

In the radiologic cohort, indications for cross-sectional imaging included determination or further characterization of a suspected cause of biliary obstruction (37.4%), presence of persistent jaundice and/or liver enzyme elevation despite apparently successful ERCP (17.6%), ruling out suspected adverse events following cholecystectomy (12.8%), ruling out Mirizzi syndrome queried during ERCP (11.8%), investigation of presentations involving other organ systems not including abdominal pain (9.6%), initial or further characterization of the biliary tree following failed or limited ERCP (5.9%), and others (4.8%). Of these patients, 148 (79.1%) had no abnormalities on cross-sectional imaging (CT or MRI) obtained within 14 days of ERCP, while 39 (20.9%) had one or more imaging findings typically associated with acute pancreatitis. Among these patients, 37 (19.8%) had evidence of pancreatic edema, inflammation, and/or fat stranding, 22 (11.8%) had incidental findings of peri-pancreatic fluid collections, 4 (2.1%) had enlargement of the gland, and only two patients (1.1%) had incidental findings of necrosis without any associated abdominal pain.

### 3.2. Predictors of Incidental Biochemical or Radiologic Alterations

Adjusted odds ratios (AORs) of incidental biochemical and radiologic findings as well as PEP following ERCP are presented in Table 3. The performance of any intraprocedural pancreatic contrast injection was associated with higher odds of asymptomatic lipasemia 2–3× ULN (AOR 7.22; 95% confidence intervals, CI, 1.13 to 46.02), as was the use of the double-wire technique (AOR 15.74; 95% 1.15 to 214.74). Placement of a common bile duct (CBD) stent was associated with lipasemia >3× ULN (AOR 4.19; 95% CI 1.37 to 12.77). The occurrence of greater than 10 cannulation attempts during ERCP was associated with the presence of one or more radiologic finding(s) (AOR 33.95; 95% CI 1.64, to 704.13), while trainee involvement and the placement of a CBD stent both trended toward significant associations (AOR 4.39; 95% CI 0.84 to 23.09 and AOR 8.10; 95% CI 0.95 to 68.95, respectively).

## 4. Discussion

In this study, we examined the incidence and predictors of incidental biochemical and radiologic pancreatic alterations in patients who underwent uncomplicated ERCP. Between 20 and 30% of patients in our cohort were found to have some evidence of pancreatic disturbance either biochemically and/or radiologically after ERCP, despite an absence of clinical symptoms or signs of acute pancreatitis or other post-ERCP AEs. Some peri-procedural parameters were identified to be associated with such biochemical and radiologic changes that are consistent with previously identified predictors of PEP (i.e., increasing cannulation attempts and performance of a pancreatogram or insertion of pancreatic guidewires) [3,14].

Although asymptomatic and inconsequential pancreatic enzyme elevation after ERCP is widely purported in the endoscopy literature, precise estimates of this phenomenon are lacking. Furthermore, the expected radiologic alterations after uncomplicated ERCP are completely unknown. This methodologically rigorous study provides clinically valuable information that is important for all practitioners who partake in the post-procedural care of patients having undergone ERCP. Given the important AE profile associated with ERCP [3], there is generally a low threshold for physicians to formally investigate any post-procedural symptoms following ERCP, especially abdominal pain. However, the findings of this study suggest that in the absence of any new or worsening abdominal pain (or abdominal pain characteristic of acute pancreatitis) following ERCP, the routine measurement of pancreatic enzymes is unnecessary, and it can potentially be misleading and harmful. Similarly, encountering incidental findings of pancreatic inflammation on cross-sectional imaging following ERCP should not prompt treatment if the patient does not have clinical symptoms of pancreatitis. 

Given that approximately one-quarter of patients in our cohort experienced a degree of asymptomatic lipase elevation following ERCP, with a similar proportion being observed to have one or more pancreatic alterations suggestive of inflammation on imaging, these patients are at risk of being misclassified as having had PEP, or worse, being subsequently treated as having acute pancreatitis, which is not without risks [15]. Although misclassification alone is arguably less consequential, downstream sequelae of inappropriate medical diagnoses portend psychosocial, medicolegal, and healthcare utilization consequences as well as biasing quality performance audits [9,16]. 

The proposed mechanisms of PEP are complex and incompletely understood. One theory is that epithelial injury to the pancreatic duct (PD) can result from hydrostatic or allergic injury from contrast or from direct injury from cannulation [17]. Another is that edema or thermal injury from cannulation or sphincterotomy of the anatomically adjacent common bile duct (CBD) can result in mechanical obstruction at the level of the papilla [17]. Whatever the mechanism(s), these changes result in the activation of pancreatic proteolytic enzymes and initiation of the inflammatory cascade in acute pancreatitis [18]. Based on the nature of the procedure, it is likely that one or more of these processes occur to some degree in many or perhaps even in most patients undergoing ERCP; what remains less clear is why some patients progress to PEP, ranging in terms of clinical severity from mild to severe, why others remain asymptomatic while having neither biochemical nor radiographic evidence of pancreatic injury, and why others are asymptomatic but have some incidental evidence of subclinical injury. We recognize that while our clinical approach to these patients is often simplified to a binary one (i.e.,: PEP or no PEP), the reality is more likely that post-ERCP changes in the pancreas represent a spectrum of involvement. What is ultimately important in the post-ERCP population is (a) identifying, treating, and monitoring patients with clinically significant pathology and (b) avoiding the routine ordering of unnecessary and potentially misleading laboratory or imaging tests.

The overall usage rate of rectal indomethacin in our study was arguably suboptimal at 41%, given the established benefits of this modality on PEP prevention [19] and accompanying guidance regarding its use in unselected ERCP patients [20]. However, this usage is likely representative of real-world practice patterns. For example, in a 2020 US study of 26 healthcare systems, the usage of rectal indomethacin was under 50% even in high-risk patients [21]. A separate 2020 survey of endoscopists identified similarly low rates of anti-inflammatory usage at 40% for average-risk patients and 60% for high-risk patients [22]. Thus, although we feel our cohort is representative of real-world practices, this could still potentially affect the generalizability of our findings.

Our study employed a prospective ERCP registry utilizing real-time third-party observation with multiple granular data points [13] to achieve its planned aims. To mitigate the risk of bias as much as possible, we established strict eligibility criteria that strived to exclude all symptomatic patients to characterize the incidence of purely incidental abnormalities in pancreatic biochemistry or imaging parameters. Each imaging study was carefully reviewed to ensure these criteria were met. Despite this, there are limitations to our study design and execution. Firstly, although prospectively collected data were used, our study was a single-center retrospective analysis of these data; therefore, selection bias remains a possibility despite our every effort to mitigate it by granular a priori definitions of exclusion/inclusion criteria. Specifically, we excluded all patients with pre-existing lipase elevations and those with non-routine bloodwork performed. Secondly, with these and other rigorous eligibility criteria in place, we could have arguably over-excluded patients from our cohort and, consequently, this could have resulted in our findings being underestimates of true incidences. 

Thirdly, our study primarily aimed to describe the incidence of biochemical and/or radiologic pancreatic abnormalities following ERCP, and it was relatively underpowered for our secondary aim, which was to assess potential predictors of these abnormalities. Future multi-center prospective studies would be beneficial in further elucidating these predictors. Fourthly, it is most common at our center to measure lipase (rather than amylase), which is appropriate given its higher sensitivity and equivalent specificity for acute pancreatitis [23]; however, our results are therefore not generalizable to centers where amylase is preferentially measured. Furthermore, although we adjusted for underlying comorbidities in our modelling, we did not adjust for acute illness in the peri-procedural period, which could have been an important confounder. Finally, our study population exclusively included inpatients, which represents an important source of potential selection bias. This is especially true when one considers that inpatients are more acutely ill, potentially more comorbid, and more prone to peri-procedural hypovolemia and/or acute kidney injury, which could all differentially affect our outcomes. Therefore, it is unclear whether our results are generalizable to outpatients, and future studies are required to investigate this population.

In conclusion, in an analysis of a prospective ERCP study, rates of incidental biochemical and radiologic pancreatic abnormalities suggestive of inflammation following ERCP are described. Stakeholders responsible for the post-procedural care of ERCP patients should be aware of our findings to minimize misclassification, overtreatment, and potential patient apprehension. Future prospective multi-center studies are required to better delineate predictors of these incidental abnormalities, their relationship, if any, to the culmination of true post-ERCP pancreatitis and to identify the degree of healthcare utilization burden and deviation from value-based care. 

## Figures and Tables

**Table 1:** Demographic, clinical and procedural details for patients with and without asymptomatic lipasemia after ERCP.

Parameters	No Lipasemia (n = 478)	Lipasemia 1–2× ULN (n = 81)	Lipasemia 2–3× ULN (n = 26)	Lipasemia >3× ULN (n = 61)	*p*-Value
Mean age (SD)	59.0 (19.1)	60.5 (18.5)	56.3 (16.0)	58.9 (19.4)	0.65
Sex, % female (n)	47.3 (226)	45.7 (37)	50.0 (13)	60.7 (37)	0.34
Mean Charlson Comorbidity Index (SD)	3.1 (2.7)	3.0 (2.5)	2.7 (2.9)	3.2 (2.8)	0.88
Indication CBD stones, % (n) Cholangitis, % (n) Malignant biliary obstruction, % (n) Pancreatic indication(s), % (n) All others, % (n)	65.3 (312) 10.5 (50) 8.8 (42) 0.6 (3) 14.9 (71)	60.5 (49) 11.1 (9) 12.3 (10) 0.0 (0) 16.0 (13)	69.2 (18) 3.8 (1) 19.2 (5) 3.8 (1) 3.8 (1)	67.2 (41) 14.8 (9) 4.9 (3) 1.6 (1) 11.5 (7)	0.76
Trainee endoscopist involved, % (n)	71.6 (342)	66.7 (54)	61.5 (16)	67.2 (41)	0.56
Prior ERCP, % (n)	20.3 (97)	18.5 (15)	3.9 (1)	16.4 (10)	0.11
Pancreatogram performed, % (n)	2.3 (11)	3.8 (3)	19.3 (5)	6.5 (4)	<0.001
PD cannulation, % (n)	18.4 (86)	28.8 (23)	50.0 (13)	39.0 (23)	<0.001
Double wire technique utilized, % (n)	11.4 (53)	28.8 (23)	45.8 (11)	27.6 (16)	<0.001
Sphincterotomy performed, % (n)	80.1 (375)	81.0 (64)	70.8 (17)	73.7 (42)	0.61
Balloon sphincteroplasty performed, % (n)	20.9 (99)	21.3 (17)	15.4 (4)	33.9 (20)	0.18
Pre-cut sphincterotomy performed, % (n)	5.7 (27)	7.4 (6)	20.0 (5)	13.6 (8)	<0.001
Needle-knife papillotomy performed, % (n)	6.1 (29)	6.2 (5)	12.0 (3)	15.3 (9)	<0.001
Cannulation attempts 1 or 2, % (n) 3 to 5, % (n) 6 to 10, % (n) >10, % (n)	56.4 (246) 27.1 (118) 9.4 (41) 7.1 (31)	47.1 (33) 31.4 (22) 8.6 (6) 12.9 (9)	40.0 (8) 20.0 (4) 25.0 (5) 15.0 (3)	29.6 (16) 37.0 (20) 25.9 (14) 7.4 (4)	<0.001
CBD stent(s) placed, % (n)	21.4 (101)	35.8 (29)	36 (9)	35.6 (21)	0.002
PD stent(s) placed, % (n)	6.9 (33)	18.5 (15)	26.9 (7)	14.8 (9)	<0.001
Mean cannulation time in minutes (SD)	4.7 (6.7)	6.3 (7.7)	6.7 (7.0)	6.6 (6.8)	<0.001
Overall procedure time in minutes (SD)	20.6 (13.9)	22.7 (13.9)	25.6 (13.8)	27.1 (15.2)	<0.001
Pre-procedural indomethacin given, % (n)	41.8 (200)	33.3 (27)	34.6 (9)	49.2 (30)	0.38
Mean lipase level within 24 h of ERCP (SD) *	36.4 (18.6)	117.3 (23.5)	196.4 (21)	1037.8 (890.8)	<0.001

* Highest value of lipase reported as ‘>3000 U/L’. ULN, upper limit of normal; SD, standard deviation; ERCP, endoscopic retrograde cholangiopancreatography; PD, pancreatic duct; CBD, common bile duct.

**Table 2:** Demographic, clinical and procedural details for patients with and without cross-sectional imaging findings consistent with acute pancreatitis following ERCP.

Parameters	No Imaging Findings Consistent with AP (n = 148)	Imaging Findings Consistent with AP (n = 39)	*p*-Value
Mean age (SD)	63.6 (16.4)	60.4 (13.1)	0.26
Sex, % female (n)	39.2 (58)	25.6 (10)	0.17
Mean Charlson Comorbidity Index (SD)	3.5 (2.8)	3.1 (2.6)	0.37
Indication CBD stones, % (n) Cholangitis, % (n) Malignant biliary obstruction, % (n) Pancreatic indication(s), % (n) All others, % (n)	31.8 (47) 13.5 (20) 28.4 (42) 0.0 (0) 26.4 (39)	33.3 (13) 5.1 (2) 20.5 (8) 5.1 (2) 35.9 (14)	0.08
Trainee endoscopist involved, % (n)	67.6 (100)	66.7 (26)	>0.99
Prior ERCP, % (n)	29.9 (44)	33.3 (13)	0.83
Pancreatogram performed, % (n)	5.4 (8)	15.8 (6)	0.04
PD cannulation, % (n)	11.3 (15)	16.1 (5)	0.54
Double wire technique utilized, % (n)	66.2 (88)	53.6 (15)	0.30
Sphincterotomy performed, % (n)	19.4 (28)	9.1 (3)	0.21
Balloon sphincteroplasty performed, % (n)	9.0 (13)	26.5 (9)	0.01
Pre-cut sphincterotomy performed, % (n)	8.3 (12)	8.8 (3)	>0.99
Needle-knife papillotomy performed, % (n)	11.3 (15)	16.1 (5)	0.54
Cannulation attempts 1 or 2, % (n) 3 to 5, % (n) 6 to 10, % (n) >10, % (n)	55.9 (62) 20.7 (23) 13.5 (15) 9.9 (11)	26.9 (7) 26.9 (7) 7.7 (2) 38.5 (10)	0.002
CBD stent(s) placed, % (n)	37.8 (54)	50.0 (16)	0.28
Metal CBD stent placed, % (n)			
PD stent(s) placed, % (n)	6.8 (10)	2.6 (1)	0.46
Mean cannulation time in minutes (SD)	4.5 (5.6)	8.7 (11.7)	0.09
Overall procedure time in minutes (SD)	24.2 (14.7)	34.0 (22.6)	0.009
Pre-procedural indomethacin given, % (n)	30.4 (45)	28.2 (11)	0.94
Mean post-ERCP day of cross-sectional imaging, % (n)	4.7 (3.5)	3.5 (3.2)	0.05
Findings on cross-sectional imaging, % (n) Pancreatic enlargement, % (n) Pancreatic edema, inflammation, or fat stranding % (n) Pancreatic necrosis, % (n) Peri-pancreatic fluid collection, % (n)	N/A	10.3 (4) 94.5 (37) 5.1 (2) 56.4 (22)	N/A

AP, acute pancreatitis; SD, standard deviation; ERCP, endoscopic retrograde cholangiopancreatography; PD, pancreatic duct; CBD, common bile duct.

**Table 3:** Adjusted odds ratios of incidental biochemical and radiologic findings after ERCP.

Parameters	AOR of Lipasemia 1–2× ULN	AOR of Lipasemia 2–3× ULN	AOR of Lipasemia >3× ULN	AOR of 1 or More Imaging Findings
Increasing age (each additional year)	1.01 (0.99, 1.03)	1.02 (0.98, 1.06)	0.98 (0.96, 1.00)	0.97 (0.93, 1.01)
Female sex (versus male sex)	1.14 (0.64, 2.04)	0.96 (0.30, 3.01)	0.68 (0.33, 1.38)	1.99 (0.46, 8.59)
Charlson Comorbidity Index 4 or higher (versus 3 or lower)	0.95 (0.47, 1.94)	0.36 (0.08, 1.58)	1.56 (0.68, 3.57)	1.43 (0.32, 6.45)
Indication for biliary obstruction (versus all others)	0.96 (0.46, 2.02)	4.52 (0.50, 40.93)	1.17 (0.43, 3.20)	0.52 (0.11, 2.33)
Trainee endoscopist involved (versus none)	0.64 (0.36, 1.14)	1.44 (0.37, 5.58)	0.80 (0.38, 1.66)	4.39 (0.84, 23.09)
Pancreatogram performed (versus none)	0.78 (0.18, 3.45)	7.22 (1.13, 46.02)	1.07 (0.18, 6.26)	4.74 (0.39, 57.81)
PD cannulation (versus none)	0.76 (0.26, 2.19)	0.33 (0.03, 3.23)	0.75 (0.24, 2.35)	1.07 (0.19, 6.20)
Double wire technique utilized (versus none)	2.69 (0.69, 10.40)	15.74 (1.15, 214.74)	3.56 (0.89, 14.26)	2.08 (0.13, 34.51)
Sphincterotomy performed (versus none)	1.14 (0.51, 2.52)	0.23 (0.06, 0.91)	1.01 (0.40, 2.59)	2.64 (0.50, 13.97)
Balloon sphincteroplasty performed (versus none)	0.89 (0.43, 1.84)	0.33 (0.06, 1.70)	2.29 (1.08, 4.85)	0.49 (0.05, 4.92)
Pre-cut sphincterotomy performed (versus none)	0.95 (0.30, 3.04)	0.96 (0.12, 7.90)	0.94 (0.26, 3.38)	2.53 (0.26, 24.96)
Needle-knife papillotomy performed (versus none)	0.88 (0.17, 4.43)	7.51 (0.54, 104.54)	1.40 (0.25, 7.97)	0.99 (0.02, 64.44)
>5 cannulation attempts (versus 5 or fewer)	0.69 (0.26, 1.85)	2.47 (0.43, 14.20)	1.25 (0.42, 3.73)	0.67 (0.07, 6.03)
>10 cannulation attempts (versus 10 or fewer)	1.49 (0.35, 6.28)	0.98 (0.08, 12.01)	0.11 (0.01, 0.88)	33.95 (1.64, 704.13)
CBD stent(s) placed (versus none)	1.29 (0.40, 4.16)	0.99 (0.09, 10.64)	4.19 (1.37, 12.77)	8.10 (0.95, 68.95)
PD stent(s) placed (versus none)	2.39 (0.74, 7.74)	0.81 (0.13, 5.18)	0.86 (0.23, 3.26)	0.09 (0.00, 2.96)
Increasing cannulation time (per additional minute)	1.01 (0.94, 1.09)	0.86 (0.71, 1.03)	0.98 (0.90, 1.06)	1.03 (0.86, 1.25)
Increasing procedure timeleft (per additional minute)	1.01 (0.98, 1.04)	1.01 (0.96, 1.08)	1.03 (1.00, 1.06)	1.02 (0.95, 1.09)
No pre-procedural indomethacin given (versus all others)	1.16 (0.61, 2.22)	0.93 (0.28, 3.05)	0.98 (0.45, 2.14)	1.78 (0.30, 10.59)

AOR, adjusted odds ratio; ULN, upper limit of normal; PD, pancreatic duct; CBD, common bile duct.

## Data Availability

Sharing of data will be considered depending on the request.
